# Supplementary material for: Anti-diabetic drug binding site in a mammalian KATP channel revealed by Cryo-EM
Source: eLife. 2017 Oct 24;6:e31054. doi: 10.7554/eLife.31054 (PMC5655142; doi:10.7554/eLife.31054)
Supplement: Transparent reporting form [file elife-31054-transrepform.docx]

***eLife’s* transparent reporting form**

We encourage authors to provide detailed information *within their submission* to facilitate the interpretation and replication of experiments. Authors can upload supporting documentation to indicate the use of appropriate reporting guidelines for health-related research (see [EQUATOR Network](http://www.equator-network.org/%20)), life science research (see the [BioSharing Information Resource](https://biosharing.org/)), or the [ARRIVE guidelines](http://www.plosbiology.org/article/info:doi/10.1371/journal.pbio.1000412) for reporting work involving animal research. Where applicable, authors should refer to any relevant reporting standards documents in this form.

If you have any questions, please consult our Journal Policies and/or contact us: [editorial@elifesciences.org](mailto:editorial@elifesciences.org).

**Sample-size estimation**

- You should state whether an appropriate sample size was computed when the study was being designed
- You should state the statistical method of sample size computation and any required assumptions
- If no explicit power analysis was used, you should describe how you decided what sample (replicate) size (number) to use

Please outline where this information can be found within the submission (e.g., sections or figure legends), or explain why this information doesn’t apply to your submission:

This is a single–particle cryo-EM study. The number of raw particles and particles included in the different refining steps as well as the final particles included in the final 3D reconstruction are stated in the Results, Materials and Methods sections and can be found in Table 1, Figure 1-figure supplement 1D.

Sample size and statistical methods for determining significance in difference for functional testing using Rb efflux essays and patch-clamp recordings are detailed in Materials and Methods and in the legends for Figure 7 and Figure 7-figure supplement 1. For these experiments, no power analysis was used and the sample size of ≥3 is considered adequate for performing statistical analysis using the standard methods stated in the manuscript.

**Replicates**

- You should report how often each experiment was performed
- You should include a definition of biological versus technical replication
- The data obtained should be provided and sufficient information should be provided to indicate the number of independent biological and/or technical replicates
- If you encountered any outliers, you should describe how these were handled
- Criteria for exclusion/inclusion of data should be clearly stated
- High-throughput sequence data should be uploaded before submission, with a private link for reviewers provided (these are available from both GEO and ArrayExpress)

Please outline where this information can be found within the submission (e.g., sections or figure legends), or explain why this information doesn’t apply to your submission:

This is a single–particle cryo-EM study. The number of raw particles and particles included in the different refining steps as well as the final particles included in the final 3D reconstruction are stated in the Results, Materials and Methods sections and can be found in Table 1, Figure 1-figure supplement 1D.

Biological repeats for experiments shown in Figure 7 and Figure 7-figure supplement 1 are as described in the corresponding figure legend.

**Statistical reporting**

- Statistical analysis methods should be described and justified
- Raw data should be presented in figures whenever informative to do so (typically when N per group is less than 10)
- For each experiment, you should identify the statistical tests used, exact values of N, definitions of center, methods of multiple test correction, and dispersion and precision measures (e.g., mean, median, SD, SEM, confidence intervals; and, for the major substantive results, a measure of effect size (e.g., Pearson's r, Cohen's d)
- Report exact p-values wherever possible alongside the summary statistics and 95% confidence intervals. These should be reported for all key questions and not only when the p-value is less than 0.05.

Please outline where this information can be found within the submission (e.g., sections or figure legends), or explain why this information doesn’t apply to your submission:

Software and algorithms used for processing of the single particle data are described in Materials and Methods, Key resources table, Table 1, and Figure 1-figure supplement 1.

Statistical tests, sample size, mean and SEM of functional tests used in experiments presented in Figure 7 and Figure 7-figure supplement 1 are described in the corresponding figure legends.

(For large datasets, or papers with a very large number of statistical tests, you may upload a single table file with tests, Ns, etc., with reference to sections in the manuscript.)

**Group allocation**

- Indicate how samples were allocated into experimental groups (in the case of clinical studies, please specify allocation to treatment method); if randomization was used, please also state if restricted randomization was applied
- Indicate if masking was used during group allocation, data collection and/or data analysis

Please outline where this information can be found within the submission (e.g., sections or figure legends), or explain why this information doesn’t apply to your submission:

This is a single-particle cryoEM study and the group allocation information does not apply.

**Additional data files (“source data”)**

- We encourage you to upload relevant additional data files, such as numerical data that are represented as a graph in a figure, or as a summary table
- Where provided, these should be in the most useful format, and they can be uploaded as “Source data” files linked to a main figure or table
- Include model definition files including the full list of parameters used
- Include code used for data analysis (e.g., R, MatLab)
- Avoid stating that data files are “available upon request”

Please indicate the figures or tables for which source data files have been provided:

Source data of the 3D reconstruction and model will be deposited into the Protein data bank upon acceptance of the manuscript.
